# Nuclear Transport Deficits in Tau-Related Neurodegenerative Diseases

**DOI:** 10.3389/fneur.2020.01056

**Published:** 2020-09-25

**Authors:** Lisa Diez, Susanne Wegmann

**Affiliations:** German Center for Neurodegenerative Diseases, Berlin, Germany

**Keywords:** tau protein, Alzheimer's disease, neurodegenerative diseases, nuclear pore complex, nucleocytoplasmic transport

## Abstract

Tau is a cytosolic microtubule binding protein that is highly abundant in the axons of the central nervous system. However, alternative functions of tau also in other cellular compartments are suggested, for example, in the nucleus, where interactions of tau with specific nuclear entities such as DNA, the nucleolus, and the nuclear envelope have been reported. We would like to review the current knowledge about tau–nucleus interactions and lay out possible neurotoxic mechanisms that are based on the (pathological) interactions of tau with the nucleus.

## Introduction

Tau is a predominantly neuronal protein and, as a representative of the microtubule-associated protein family (1), contributes to the stabilization of microtubules (MT) and the modulation of their dynamics (2, 3). In neurodegenerative diseases such as Alzheimer's disease (AD), frontotemporal dementia with Parkinsonism on chromosome 17, Pick's disease, and others, intraneuronal aggregates of hyperphosphorylated tau are a hallmark pathological feature (4, 5), and their appearance correlates well with neuronal loss in these diseases (6–8). The filamentous tau aggregates found in human brain are amyloid-like and have a high β-sheet content; however, the architecture of their fibril core differs to some degree between aggregates from different tauopathies (9–12). *In vitro* aggregation of tau into filamentous aggregates can efficiently be induced by polyanionic co-factors such as heparin (13, 14), RNA (15), and arachidonic acid (16). However, small soluble oligomeric tau species also appear to contribute to synaptic dysfunction and cell death in tauopathies (17, 18) and are considered to mediate neurotoxicity before neurofibrillary tangle (NFT) formation (19, 20).

Monomeric tau is a highly soluble, intrinsically disordered protein that comprises four different major domains: the acidic N-terminal half (projection domain) projects from the MT surface and the proline-rich domain, which harbors a SH3-protein binding site (21, 22); the function(s) of these parts of the tau protein are rather uncertain, and they thus may play a role in alternative tau functions. The basic repeat domain containing four ~30-amino-acid-long pseudorepeats is responsible for MT binding (23–26) and aggregation of tau (25, 27). The role of the shorter C-terminal end is unknown. In the human central nervous system (CNS), tau exists in six isoforms, which carry three or four pseudo-repeats in the repeat domain (3R and 4R isoforms) and zero, one, or two repeats in the N-terminal half (0N, 1N, or 2N isoforms) and are generated by the alternative splicing of exon 2, exon 3, and exon 10 in a 6-kb mRNA transcript.

The amino acid sequence of tau harbors 85 putative phosphorylation sites (28, 29) and various sites for other post-translational modifications (PTMs) such as acetylation, methylation, and glycosylation (30, 31), which enable a complex regulation of tau's binding to MTs and its other functions (32). Phosphorylation is by far the most studied PTM of tau, also for nuclear tau.

Tau is highly abundant in axons of the CNS (33), but under stress and in pathological conditions, it can also be found in the soma, the dendrites, and the nucleus (34). This unusual cellular distribution of tau enables condition- and subcellular environment-dependent interactions (35, 36), for example, with the nucleus.

The first indication of nuclear tau, in the form of short paired helical filaments, came from transmission electron microscopy of AD frontal lobe sections by Metuzals et al. (37), and until today neither a physiological nor a pathological role of nuclear tau is clearly established. Interactions of tau with the outer neuronal envelope (NE) were recently suggested to induce deficits in RNA and protein transport in and out of the nucleus (38, 39). Regulated nucleocytoplasmic transport (NCT) of cellular biomolecules—such as transcription factors, mRNA and ribosomal RNA, and nuclear and cytosolic proteins—is essential for major principles of cell survival and function, for example, signal transduction, stress response, and proteostasis (40–42). In the recent years, defective neuronal NCT has been described in different neurodegenerative disorders (NDDs) like amyotrophic lateral sclerosis (ALS), frontotemporal dementia (FTD) (43, 44), Huntington's disease (HD) (45, 46), and AD (38).

In this review, we summarize the interactions of tau with the nucleus and discuss their potential role in pathology. After introducing known and conceivable interactions of tau with the nucleus—both of intranuclear as well as extranuclear tau, either direct or indirect—we will summarize the findings of NCT impairments in other NDDs and normal aging, aiming to gain an overall mechanistic insight for NCT disruptions as a potential culprit and therapeutic target in neurodegenerative diseases.

## How Does Tau Interact With the Nucleus?—Known and Conceivable Points of Interaction

### Nuclear Tau Isoforms and Post-translational Modifications

In the last three decades, several interactions of tau with the nucleus were reported, and potential nuclear functions of tau were suggested. Early indications of tau in the nucleus came from Binder and colleagues who showed, by immunohistology, that tau can be found in neuronal nuclei in the human brain—both in healthy controls and in AD patients (47). Following this observation, a number of studies showed tau in the nucleus of neuronal cells [e.g., human neuroblastoma (48–50) and rat cells (51)] in primary mouse neurons (52), in the mouse brain (53–55), and also in non-neuronal cell lines (e.g., fibroblasts and lymphocytes) (56, 57). In the nucleus, tau seems to be predominantly localized to the nucleolus (49, 56, 58).

In adult mice, which express 4R but not 3R tau, isoform-specific tau antibodies revealed that 1N4R tau is enriched in the nuclear-enriched fraction of brain lysates (54). It remains unclear how 1N4R tau gets into the nucleus because none of the CNS tau isoforms carries a (known) nuclear localization signal (NLS) that would enable its transport through nuclear pores into the nucleoplasm. For now we can only speculate about scenarios that would explain the occurrence of tau protein in the nucleus: specific PTMs, e.g., phosphorylation or SUMO-lation, alter the ability of transcription factors to interact with nuclear transport factors and enable their nuclear import (59, 60); a similar PTM-based mechanism could facilitate tau protein transport from the cytosol through the nuclear pore into the nucleoplasm. Another possibility could be nuclear targeting of tau transcripts, which could direct tau mRNA into the nucleus, where local transcription could produce tau protein. In fact, it has been suggested that the majority of nuclear tau may be produced by a less abundant 2-kb transcript that contains the entire tau coding region (61). Transcription of both the 2- and 6-kb tau mRNA starts at the same unique site at the start of exon 1; however, the two transcripts utilize two alternate polyadenylation sites downstream of exon 14 (62).

Both phosphorylated tau and tau dephosphorylated at certain residues have been reported in the nucleus (47, 49, 63). By immunofluorescence imaging and western blot using an antibody specific for the absence of phosphate groups at residues S195/198/199/202 (Tau-1 antibody), the majority of nuclear tau appears to be dephosphorylated, at least at these residues (48, 61). A pronounced accumulation of Tau-1 positive tau in the nucleus was observed upon acute oxidative and heat stress both *in vitro* and *in vivo* (52). Tau colocalizing with the nucleolus also seems to be mostly non-phosphorylated at residues S195/198/199/202 (Tau-1 positive) (56). However, in models of tau-induced neurodegeneration, phosphorylated nuclear tau appears to be associated with neurodegeneration (28, 64–66). In tau overexpressing SH-SY5Y cells—a model manifold used to study nuclear tau—phospho-site-specific tau antibodies revealed nuclear tau phosphorylated at specific sites such as S205, T181, T212, S404, and others (29, 67).

On a general note, the detection of nuclear tau in the mentioned studies relies mainly on the use of antibodies—for example, Tau-1 (non-P S195/198/199/202/) and AT8 (pS202/205)—that may show some unknown cross-reaction with other nuclear proteins. Therefore, more complementary proteomics studies, as that's performed by Ulrich et al. (29), will be needed to clarify which tau isoforms and PTMs occur in the nucleus. Furthermore, the biochemical detection of nuclear tau is usually based on cellular fractionation protocols, in which nuclear-enriched fractions are analyzed for their tau content; from these data, it remains unclear whether tau is present in the nucleoplasm or is associated with the inside or the outside of the nuclear envelope. As of now, it also remains unclear how nuclear tau (PTMs and isoforms) differs between cell types, differentiation state, and host species and which nuclear tau species may be relevant for neuronal function in the human brain.

### Intranuclear Tau: Interactions of Nuclear Tau With Intranuclear Components

#### Tau–DNA Interactions

Different microtubule-associated proteins, such as tau and MAP2, were shown to interact with DNA (68, 69). Tau–DNA interactions appear to be, to some degree, nucleic acid sequence-specific for single-stranded DNA, with some preference for GC-rich regions, whereas some studies identified no or little sequence specificity for tau binding to double-stranded DNA (70); preferential binding of tau to AG-rich sequences was also reported (55). For both ssDNA and dsDNA, tau binding was reported to be facilitated by the minor DNA groove *via* electrostatic interactions (2, 71, 72), similar to the DNA binding mechanism of histones and other chromatin architectural proteins (73–75), which may suggest a potential chaperone-like function of tau for DNA folding (28, 75).

By nuclear magnetic resonance (NMR) spectroscopy, the tau binding motif for DNA was assigned to the C-terminal half of the proline-rich region and repeat 2 in the repeat domain of human tau (76). Both of these regions in tau are commonly phosphorylated in physiological and pathological conditions (77), which indicates a potential role of phosphorylation (or other PTMs in these regions) for the regulation of tau binding to DNA. Interestingly, the tau-interacting regions in genomic DNA of mouse primary neurons were found to be distributed across different chromosomes and between genic and intergenic regions as shown by chromatin immunoprecipitation with the anti-tau antibody Tau-1 (55). Heat stress, which induces tau phosphorylation at certain epitopes and de-phosphorylation at others, induced an increase in nuclear tau and a global dissociation and redistribution of tau on chromatin (55). Interestingly, hypothermia also induces tau phosphorylation by GSK3β and CDK5 (78) but it is—to our knowledge—not known whether it changes the abundance of tau in the nucleus.

Heat-stress-induced nuclear tau is unphosphorylated at sites T212, T231, T235, S262, S356, S396, and S404 (52), which indicates that the binding of tau to negatively charged DNA could be regulated by phosphorylation, similar to the MT binding of tau. This idea is supported by *in vitro* NMR and surface plasmon resonance (SPR) measurements that show a pronounced reduction of DNA binding ability of phosphorylated compared to unphosphorylated recombinant protein (75, 76). In addition to electrostatic interactions, hydrophobic interactions were found to further stabilize tau–DNA interactions (75, 76).

#### Suggested Functions of Tau–DNA Interactions

The physiological and the pathological roles of tau binding to DNA are still unclear, and different potential functions have been suggested. For example, the binding of tau to DNA seems to induce a bending and associated conformational changes in the DNA backbone, similar to what is commonly observed for proteins that physically protect DNA from damage (28). Accordingly, primary mouse neurons that are lacking tau show a higher rate of DNA damage in Comet assays, which detects *inter alia* single- and double-stranded DNA breaks (79). This suggests that tau can protect DNA against oxidative and hyperthermic stress, which indicates that tau may function as a protector of genomic integrity under stress conditions (52). In hypothermic mice, which show a transient increase in reactive oxygen species in the brain, the presence of tau also protected against heat shock, suggesting a role of tau in modulating double-strand break DNA repair (53). Recently, tau's involvement in DNA damage response was further defined by Sola et al., who used tau-knockout human neuroblastoma cells (tau-KO SH-SY5Y) to shown that tau-deficient cells are less sensitive to DNA damage-induced apoptosis mediated by p53 modulation (80). A protective role of tau was also suggested on the chromatin level: using immunostainings of fibroblasts from FTD patients carrying the tau P301L mutation, Rossi et al. found chromosome aberrations as well as chromatin and spindle abnormalities and concluded that tau could promote chromosome stability (67, 81). Changes in chromatin and in gene expression in response to tau were also found in other studies (82, 83). For example, the clustering of histone H3 trimethylated at lysine 9 (H3K9me3) and heterochromatin protein 1α (HP1α), markers of heterochromatic DNA is disrupted in tau-deficient mice, indicating that tau may be involved in the epigenetic regulation of gene expression (84). Frost et al. provided a link between mutant tau expression, oxidative stress, and heterochromatin relaxation: upon human mutant tau P301L expression in *Drosophila*, genes that were normally silenced by heterochromatin (such as *Ago3*, the *Drosophila* homolog of human *PIWIL1*) had an increased expression, and neurons showed cell cycle reactivation, a condition that can drive the apoptosis of post-mitotic neuronal cells (82).

A structure-building role of tau in the nucleus was implicated by Sjöberg et al., who reported the binding of tau to pericentromeric DNA in human fibroblasts, lymphoblasts, and HeLa cells and suggested the involvement of tau in nucleolar organization (85). With the nucleolus being the center of ribosomal DNA (rDNA) metabolism and ribosomal complex formation, tau could thus control the rate of ribosome assembly and thereby influence RNA translation (86) or “heterochromatize” (=silence) rRNA genes as observed for other heterochromatin-associated proteins (85). SH-SY5Y cells also showed that tau associates with nucleolar TIP5, a key factor in heterochromatin stability and rDNA transcriptional repression, suggesting a role of tau in rDNA silencing (50).

In summary, intranuclear tau may directly protect DNA integrity, participate in DNA repair mechanisms, be involved in gene regulation, or help to control ribosomal gene translation and assembly.

#### Intranuclear Tau in Pathology

It has been shown that phosphorylation reduces the nuclear localization of tau (63, 87) and its ability to bind and protect DNA (29, 75, 76, 88), suggesting a potentially harmful loss of nuclear function for hyperphosphorylated tau. The overall absence of tau—and therefore also absence of nuclear tau—in tau-knockout mice has been shown to alter the chromatin arrangement and render neurons more vulnerable to heat stress (53). An increase in cytosolic tau phosphorylation may also be upstream of oxidative stress-induced DNA breakage (63, 82, 89). In any case, nuclear tau alteration capable of disrupting the chromatin organization or inducing DNA damage would dysregulate neuronal gene expression (82), which ultimately could cause neuronal death. However, it is yet unclear to what extent and how intranuclear tau contributes to neurotoxicity and if disease-associated tau mutations contribute to nuclear alterations.

### Extranuclear Tau: Interaction of Cytoplasmic Tau With the Nuclear Envelope

In NDDs like AD and tauopathies, a substantial amount of tau is found in the somatodendritic compartment where it can interact with the outside of the nucleus, the outer NE. The transport of RNA and proteins across the NE is regulated by nuclear pores and is essential for many cellular functions. In the following, we introduce the architecture and the function of nuclear pores, and then we will review what is known about interactions between cytosolic tau and the nucleus, which can be of either direct or indirect nature.

#### Nuclear Pore Complexes and Nucleocytoplasmic Transport

The nucleus is enclosed by the NE, a double lipid bilayer that separates the nuclear interior from the cytoplasm. The outer nuclear membrane is continuously connected to the endoplasmic reticulum membrane system. The inner nuclear membrane is lined with the nuclear lamina, a fibrous meshwork of lamin proteins that provides structural support to the NE (90) and also serves as a scaffold for chromatin attachment (91). The linker of nucleoskeleton and cytoskeleton (LINC) protein complex contributes to nuclear stability and positioning by physically linking the lamin-rich nucleoskeleton to the cytosolic cytoskeleton that comprises *inter alia* actin microfilaments or microtubules (92, 93). To allow for controlled macromolecular trafficking of proteins and RNA between the nuclear interior and the surrounding cytoplasm—a basic process essential for cellular protein homeostasis—the NE is homogenously “perforated” by nuclear pores, which are built by nuclear pore complexes (NPCs) (94, 95). NPCs are among the largest cellular macromolecular assemblies: vertebrate NPCs, for example, have a molecular weight of ~120 MDa (96). Multiple copies of around 30 different proteins, called nucleoporins (Nups), constitute the building blocks of the NPC, yielding a total of ~500–1,000 proteins (97). The overall structure of the NPC is conserved across different cell types; however, studies indicate that cells may express unique combinations of NUPs to generate NPCs with specialized functions (98). The center of the nuclear pore is built by a complex cylindrical structure that displays a rotational symmetry of eight subunits surrounding a central tube, through which the nucleoplasm is connected to the cytoplasm and where the exchange of macromolecules between these two cellular compartments takes place (99). From the central pore, largely unstructured, filamentous proteins extend into both the cytoplasmic and the nuclear spaces. On the nuclear side of the pore, eight protein filaments form a basketlike structure by joining into a distal ring (96).

The different Nups are classified regarding their function and location in the NPC (97, 99–101): (i) scaffold or coat Nups determine the structure of the nuclear and the cytoplasmic rings (e.g., Sec13, Seh1, Nup96, Nup75, Nup107, Nup160, Nup133, Nup37, Nup43, and ELYS), (ii) transmembrane Nups or pore membrane proteins (POMs) hold the NPC in position through transmembrane domains that interact with the NE (NDC1, POM210, and POM121), (iii) central channel Nups form the pore of the NPC (Nup205, Nup188, Nup93, Nup155, Nup53, Nup54, Nup58, Nup62, and Nup98), (iv) cytoplasmic ring/filament Nups are projecting into the cytoplasm from the NPC (Rae1, Nu42, Nup88, Nup214, DDX19, Gle1, and RanBP2/Nup358), and (v) nuclear ring/basket Nups are involved in the organization of the NPC cargo transport machinery by facilitating the recognition and the binding of nuclear import and export factors on the nuclear side of the NPC (Nup153, Nup50, Tpr). Of special importance to the NCT of biomolecules through the nuclear pore are the so called FG-Nups, which are central-channel Nups with intrinsically disordered domains rich in phenylalanine-glycine repeats (FG) (102–104). FG-Nups are attached to the nuclear scaffold *via* coiled-coil protein motifs in their non-FG domains, whereby their long FG-domain containing N-terminal parts extends as unstructured polypeptides into the central channel; here they create a hydrogel-like polymer brush that acts as a selectively permeable diffusion barrier for the transport of proteins and other biomolecules (95, 104–106). In their free state *in vitro*, FG-rich Nups spontaneously undergo liquid–liquid phase separation (LLPS) and form hydrogel-like droplets (107). Small nonpolar molecules and ions <40 kDa can passively co-partition into the Nup hydrogel phase and diffuse through the nuclear pore, whereas polar or bigger macromolecules have to be actively transported through the pore in an energy-dependent manner (99, 105, 108).

Intriguingly, multiple proteins that aggregate and form intracellular inclusions in NDDs with detectable NCT impairment are able to also undergo LLPS, for example, the RNA binding proteins FUS (109) and TDP-43 (44, 110), polyQ–Htt (111, 112), and also tau (113–115). One may thus suspect a (mis) functional connection between the liquid protein phase behavior of Nups and these proteinopathic hallmark proteins—e.g., due to co-phase separation, co-aggregation, or NTF loss or gain of function—which in neurodegenerative diseases could then result in NPC dysfunction with neurotoxic consequences.

Active NCT requires interactions between soluble nuclear transport receptors (NTRs) and Nups in the central channel of the NPC. The most common family of NTRs are the karyopherins, also called importins or exportins depending on their transport function into or out of the nucleus (96, 116). Cargo molecules that are supposed to be shuttled into the nucleus or exported from the nucleus are equipped with specific amino acid sequences: a nuclear localization signal (NLS) mediates the import, and a nuclear export signal (NES) mediates the export from the nucleus. Notably, proteins that need to shuttle between the nucleoplasm and the cytoplasm, such as the RNA-binding proteins TDP-43 (117) and FUS (118, 119), can carry both a NLS and a NES. The NCT of NTR-bound cargo molecules further depends on the nucleocytoplasmic gradient of RanGTP and RanGDP, with a high RanGTP concentration inside the nucleus and high cytoplasmic levels of RanGDP (95, 120, 121). If the RanGTP or the RanGDP gradient is destroyed, NCT is not possible (122). In an import scenario, NLS-cargo is bound to cytoplasmic importin-β–either directly or indirectly *via* the adaptor karyopherin importin-α–and is then shuttled through the NPC *via* hydrophobic interactions with FG-Nups (123). In the nucleus, the NLS-cargo is released when the importin transport receptor interacts with intranuclear RanGTP (124). In an export scenario, the exported NES-cargo is released into the cytoplasm upon GTP hydrolysis of RanGTP by RanGAP1, a GTPase-activating protein located on the cytoplasmic filaments of the NPC (45).

Different NPC models try to explain the molecular mechanism of nucleocytoplasmic transport. The “virtual gating/polymer brush” model suggests that non-interacting FG-Nups extend into the pore and form a polymer brush that functions as an entropic diffusion barrier on both sides of the NPC. While large macromolecules are generally hampered from NPC passage, the binding of transport receptors to the FG-repeats in central pore Nups can facilitate the translocation of their entrained cargo (104). The “selective phase/hydrogel” model anticipates the formation of a hydrogel-like molecular sieve that is formed *via* hydrophobic interactions among FG-repeats (104, 122). While smaller molecules can easily diffuse through the FG-Nup hydrogel meshwork, larger biomolecules cannot penetrate the hydrogel and are thus restrained. NTR–cargo complexes can bind to and dissolve into the FG-Nup meshwork and therefore can be translocated (125).

The regulated bidirectional transport of proteins and RNA in and out of the nucleus is important for many key cellular processes, for example, chromatin assembly, DNA metabolism, RNA synthesis and processing, signal transduction, and ribosome biogenesis. It is therefore obvious that any deregulation and impairment of the NCT can have detrimental consequences for the cell, leading to toxicity and cell death at worst (95). For instance, loss of the nuclear–cytoplasmic Ran-gradient, maintained by RanGAP1, can lead to cell death within minutes (45, 126).

Interestingly, Nups have also been shown to be involved in NCT-independent functions such as microtubule attachment to kinetochores, regulation of genome organization and gene expression, cell differentiation and development, RNA processing, and quality control (45, 96, 100). FG-Nups like Nup62, Nup153, and Nup98 are of special importance for transcription and chromatin organization (127, 128). These findings suggest that even disturbances at the level of the NPC building blocks can have vast cellular consequences.

#### Tau-Induced Irregularities of the Nuclear Membrane

In AD, ALS, FTD, and HD, pronounced irregularities and invaginations in the normally smooth neuronal NE have been identified by immunohistology and electron microscopy of post-mortem patient brain tissue (38, 39, 129–131).

In the case of tau, nuclear membrane abnormalities and clumping of nuclear pores have been observed in the nuclei of both NFT-neurons and neighboring pre-tangle (37, 132, 133). Alterations in the nuclear architecture were also observed in SH-SY5Y cells overexpressing human tau in the cytosol (134), which induced extensive lobulations in the NE and re-arrangements of the filamentous lamin nucleoskeleton. However, neither degradation of nuclear lamins nor cell death was observed in these cells. Tau-induced lamin dysfunctions were also shown *in vivo* in a *Drosophila* tau FTD-model, where they seemed to occur downstream of aberrant tau phosphorylation and led to neurotoxicity (135). Pathological tau was found to overstabilize F-actin, which led to a disruption of the LINC complex organization and thereby reduction and disorganization of lamin in neurons. As a consequence of the lamin dysfunction, relaxation of heterochromatic DNA was accompanied by subsequent DNA damage, aberrant cell cycle activation, and apoptosis (135). More recently, Frost and colleagues were able to show that the observed FTD-mutant tau-induced NE invagination can also cause a toxic accumulation of mRNA (39). Interestingly, a defective nuclear lamina and NCT impairment—similar to the NE distortions observed in neurons with tau accumulation (38, 82, 135)—occurs also in the premature aging disease Hutchinson-Gilford progeria syndrome (136), suggesting that NE distortions could be a common phenotype in neurodegenerative protein aggregation diseases and aging.

Two more indirect tau–nucleus interactions were recently presented: Autosomal-dominant FTD-tau mutations were shown to cause microtubule-mediated deformation of the nuclear membrane in human induced pluripotent stem cell (iPSC)-derived neurons (131), which resulted in defective NCT, and rod-like cytoplasmic tau aggregates at the nuclear envelope were shown to distort the nuclear membrane in striatal neurons in HD and in pre-tangle neurons in AD, and in mice expressing FTD-mutant tauP310S (37, 137, 138).

#### Direct Interactions of Tau With the Nuclear Envelope

**Figure 1:**
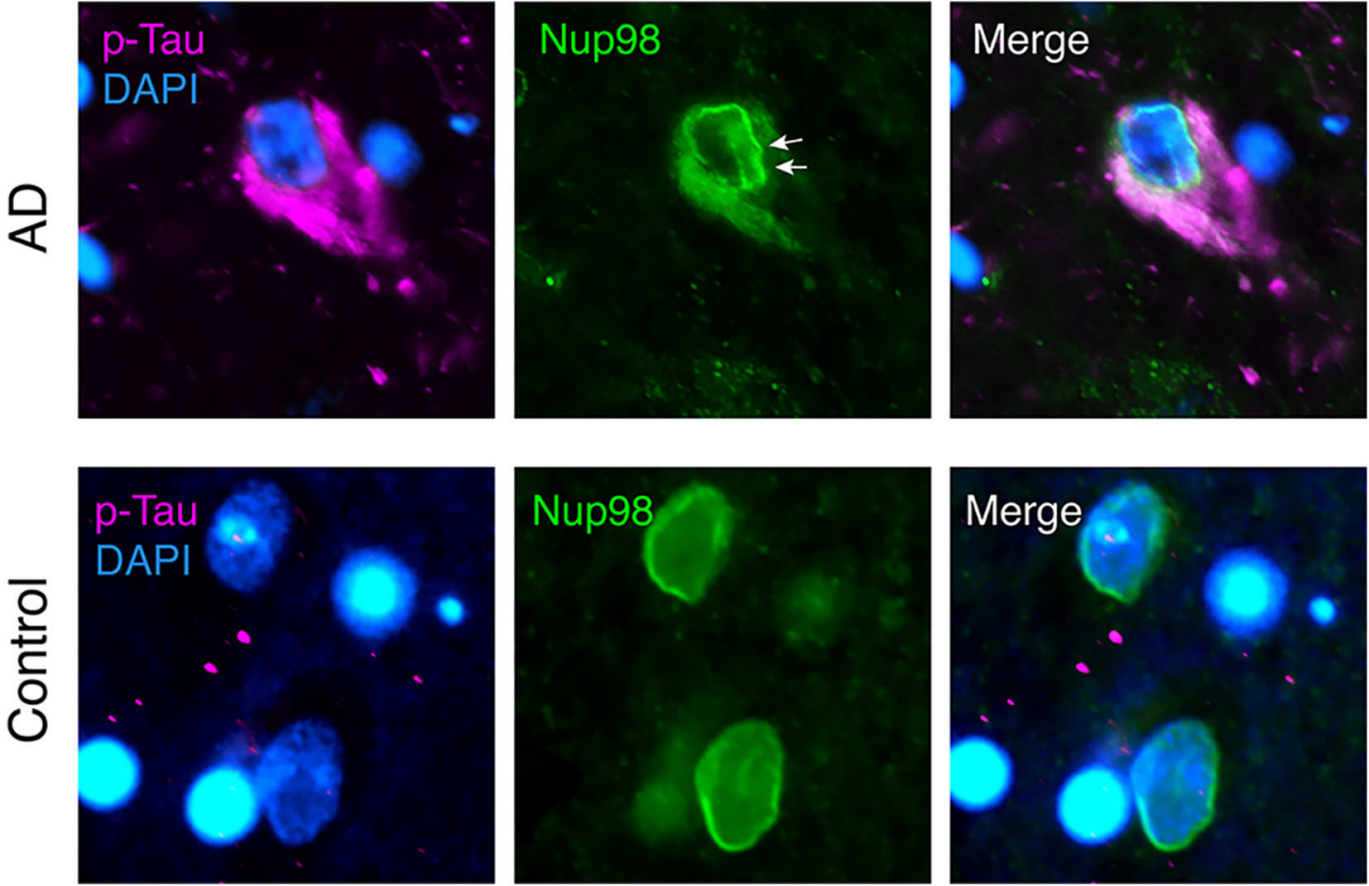
Nuclear envelope distortion and cytoplasmic mislocalization of Nup98 in neurofibrillary tangles (NFTs) of Alzheimer's disease (AD) brain. Human AD (Braak V) and age-matched control brain sections were immunolabeled for phospho-tau (magenta; p-tau mix of anti-phospho-tau antibodies, pS199, pT205, pS262, pT231, and pS409), Nup98 (green), and Dapi (blue). In AD cortex, NFTs filled with phospho-tau show a crinkled/distorted nuclear envelope (white arrows) and Nup98 accumulation in the cytosol. In control nuclei, Nup98 is localized evenly to the nuclear membrane.

Evidence for a direct interaction of cytoplasmic tau with NPCs was recently provided by Eftekharzadeh et al. (38). Hippocampal neurons in post-mortem AD brain had a distorted NE and abnormal irregular NPC distribution, and certain FG-Nups accumulated in the cytoplasm of NFT-neurons (Figure 1). In tangle-free neurons, phospho-tau accumulated at the nuclear membrane. Using SPR of recombinant proteins and co-immunoprecipitation of tau and Nup98 from human AD brain tissue, a direct interaction of tau with the FG-Nups Nup98 and Nup62 was shown. Interestingly, the C-terminal half of Nup98—one of the most abundant Nups with the highest FG content (107)—was able to trigger tau aggregation *in vitro*, suggesting a possible contribution of soluble cytoplasmic Nup98 to tau tangle formation (38). The C-terminal part of Nup98, which is usually buried in the NPC scaffold, is highly negatively charged and may therefore efficiently induce tau aggregation, similar to other polyanionic macromolecules like heparin and RNA (13–15, 139). In the same study, it was also shown that cytosolic tau can induce neuronal NCT impairments (38). In tau-overexpressing transgenic mice, primary mouse neurons, and human AD brain tissue, the presence of phosphorylated tau in the neuronal soma led to a depletion of nuclear Ran and an impairment of both nucleocytoplasmic import and export of proteins. Notably, NCT and Nup98 defects could be rescued in FTD-tau transgenic mice by reducing soluble transgenic tau, suggesting a new pathogenic mechanism, in which the somatodendritic accumulation of tau enables abnormal interactions of tau with components of the NPC and leads to NCT impairment, which is further accompanied by cytoplasmic aggregation of nucleoporins.

#### Indirect Effects of Tau on the NCT

Despite the direct interaction of tau with Nups, indirect effects of tau on the NCT have also been reported. For example, abnormal cytoplasmic accumulation of NTF2, a RanGDP transporter and key NCT factor, indicated an impaired NCT in the hippocampal neurons of AD brains (133). In another study, importin-α1 localized to “Hirano bodies”—inclusions containing actin and actin-associated proteins—in AD hippocampal neurons, whereas control brains showed a diffuse cytoplasmic localization of importin-α1 (133, 140). Notably, importin-α1 did not co-localize with NFTs or amyloid-β plaques in AD brains and not with Lewy bodies in PD brains.

The disease-associated depletion of Nups from NPCs, as suggested by the cytoplasmic sequestration of Nup98 into NFTs in AD brain (38), could deplete Nup98 from NPCs and lead to NPC disassembly and loss of function. Unspecific clogging of the nuclear pore by tau aggregates could comprise another tau–NPC interaction, leading to NCT impairment.

Another concept for a potential indirect interaction of tau with Nups is based on a study by Toda et al. (141): Nup153 associates with the transcription factor Sox2 to regulate the neural fate of neural progenitor cells (141), whereby Nup153 binding to both the 5′ and the 3′ ends of genes enables a bimodal gene regulation. Other Nups also play a role in transcription regulation (128, 142, 143). Even though the interaction of tau with Nup153 has not been investigated, disease-associated binding of tau to Nups that play a role for transcription could induce tau-mediated gene alterations in neurodegenerative diseases.

## Nct Impairment as a General Concept in Neurodegeneration?

In recent years, the disruption of neuronal NCT has been observed in different neurodegenerative protein aggregation diseases such as HD, ALS, and FTD (97, 144, 145) and recently also as an effect of tau in AD (38). It has been suggested that NCT failure is caused by pathological perinuclear protein aggregation in general [e.g., artificial β-sheets, polyQ–Htt fragments, cytoplasmic fragment of TDP-43 (146)]; however, the molecular and the cellular mechanisms as well as the downstream effects of disease-associated NCT impairment need to be further investigated in order to identify similarities and differences across diseases. Observations associated with NCT failure that are common in different protein aggregation diseases seem to be, for example, (i) the mislocalization of nuclear transport receptors and nucleoporins (45, 147–149), (ii) the mislocalization and the aggregation of RNA-binding proteins (149), and (iii) the loss of chaperone activity exhibited by certain nuclear import receptors (149, 150). In the following, we provide an overview of nucleocytoplasmic trafficking defects in neurodegenerative diseases other than AD.

### Nup Mutations Linked to NCT Impairment

Only few neurodegenerative-disease-relevant mutations have been identified within proteins of the NPC/NCT machinery. A missense mutation (Q391P) in the FG-Nup Nup62 was found in autosomal recessive infantile bilateral striatal necrosis, a fatal neurological disorder characterized by bilateral symmetric degeneration of the basal ganglia, the caudate nucleus, and the putamen (151). In ALS, two mutations in the human cytoplasmic ring Nup Gle1 were shown to cause the depletion of Gle1 from the NPC; Gle1 is essential for nuclear mRNA export (152).

### NCT in Amyotrophic Lateral Sclerosis and Frontotemporal Dementia

Most information about nuclear transport failure in neurodegeneration comes from ALS and FTD research. Accordingly, different recent review articles already cover this topic in detail (97, 122, 144, 145, 153, 154), and we therefore give only a short summary of what is known about NCT impairments in the etiology of ALS/FTD.

ALS and FTD share some clinical, neuropathological, and genetic features and therefore are classified in a common disease spectrum with likely similar neurodegenerative pathways (155). ALS is characterized by a progressive degeneration of motor neurons, which leads to increasing muscle weakness and loss of mobility. FTD, the second most frequent form of dementia, is characterized by frontal and temporal lobe degeneration, which clinically leads to social and behavioral changes (155). A common abnormality in both ALS and FTD is the mislocalization of RNA-binding proteins (RBPs) from the nucleus into the cytoplasmic aggregates in the affected neurons (122). These RBPs include the nuclear protein TAR DNA-binding protein of 43 kDa (TDP-43) and fused in sarcoma (FUS) protein (149, 156, 157); for both proteins, a nuclear loss-of-function and a cytoplasmic gain-of-toxicity are discussed (149). Indications for NPC/NCT disruption in ALS are evident from nuclear membrane irregularities and abnormal NTR distribution in motor neurons (122, 158, 159) and in neurons with cytoplasmic TDP-43 inclusions in post-mortem ALS tissue (160).

The most common genetic cause of ALS/FTD is a repeat expansion of the chromosome 9 open reading frame 72 (C9orf72) (97), which has been linked to NCT impairment on different levels. In yeast and fly models of ALS/FTD C9orf72, different Nups act as suppressors (e.g., Nup107, Nup50, and Nup98) or enhancers (e.g., Nup62 and Gle1) of C9orf72-associated cell toxicity (97, 161–163). Furthermore, RanGAP1 can directly interact with the intronic hexanucleotide (G_4_C_2_) in the C9orf72 repeat expansion (147, 164), and it accumulates in cytoplasmic punctae in the motor cortex of ALS C9orf72 patients in patient-derived iPSC neurons (147, 165).

Besides *C9orf72*, familial ALS-associated mutations in copper- and zinc-superoxide dismutase (SOD1), in TDP-43, and in FUS have been shown to cause NCT failure. For example, in transgenic mutant SOD1 mice, the subcellular redistribution of importin-β and importin-α from the nucleoplasm into the cytosol has been reported (166). Additionally, misfolding of SOD-1 can expose its normally buried NES-like sequence, which leads to exportin-1-mediated nuclear export of misfolded SOD1 (167). Cytoplasmic accumulation of Nups and RanGAP1 in stress granules was also observed in ALS-SOD1 (42, 149).

In the case of ALS/FTD TDP-43, the pathological cytoplasmic aggregation of TDP-43—an essential nuclear RNA-binding protein and splicing regulator—is associated with mislocalization and/or cytoplasmic aggregation of Nups and nuclear transport factors, with a disruption of the nuclear membrane and NPCs, and, consequently, with the reduction of nuclear protein import and mRNA export (43). By proteomic analysis, components of the NPC/NCT, predominantly FG-Nups (e.g., Nup62, Nup98, and Nup153), scaffold Nups (e.g., Nup35 and Nup93), and nuclear export factors such as Xpo5 and Nxf1 were shown to co-aggregate with pathological cytoplasmic TDP-43. Notably, TDP-43 toxicity and defective NCT function in neurons overexpressing the C-terminal fragment of TDP-43 could be rescued upon treatment with selective nuclear export inhibitors (KPT-276 and KPT-335) (43).

For the nuclear RNA-binding protein FUS, about half of the ALS/FTD mutations affect its NLS sequence, which leads to disease-associated mis-localization, stress granule formation, and aggregation of FUS in the cytoplasm (118, 153). In a *Drosophila* model of human FUS overexpression in motor neurons, neurotoxicity could be prevented by the downregulation of Nup154 (fly ortholog of human Nup155) and exportin-1 (168), supporting a role of the NCT for FUS toxicity in this model. Elsewhere it was suggested that NCT proteins (e.g., exportin-1) modulate FUS toxicity by acting on the mislocalization and the aggregation of FUS itself (122).

In conclusion, comprehensive evidence suggests that NCT dysregulation is a pathogenic driver of neurotoxicity in ALS and neurodegeneration (153).

### NCT in Huntington's Disease

Huntington's disease is caused by a CAG-repeat expansion in exon 1 of the huntingtin gene, which leads to a long polyglutamine (polyQ; *n* = 35–60+) stretch on the N-terminal end of the Huntingtin protein (Htt) (169, 170). Htt is equipped with an internal NLS and NES sequence and therefore can shuttle between the nucleus and the cytoplasm (171–173). Under disease condition, polyQ–Htt aggregates in the nucleus and the cytosol—mostly in neurons of the striatum and the cortical regions, but also in the hippocampus (169, 174, 175)—and thereby induces neurotoxicity (45, 176).

Within intracellular polyQ–Htt aggregates, FG-repeat Nups of the NPC cytoplasmic filaments (DDX19, RanBP2, and Nup214), the nuclear basket (Nup153), and the central channel (Nup62) have been identified (177). Another interactome study identified RanGAP1, nucleoporin Sec13, and the mRNA export factor Rae1 (ribonucleic acid export 1) as interaction partners of polyQ–Htt (178). Grima et al. confirmed the interaction of Nup62 and RanGap1 with intranuclear polyQ–Htt inclusions in HD transgenic mouse and *Drosophila* models, primary neurons expressing polyQ–Htt, HD patient-derived iPSC neurons, and post-mortem human HD brain regions (45). In fact, multiple NPC proteins were severely mislocalized and aggregated in the cytosol, particularly those from the cytoplasmic ring/filaments (Nup88 and Gle1) and central channel. In neurons with polyQ–Htt inclusions, both passive and active NCT and the Ran gradient were disrupted. Importantly, treatment with the small molecule nuclear export inhibitor KPT-350 as well as overexpression of RanGAP1 were both able to restore the nucleocytoplasmic Ran gradient (45), rescue cell death, and increase cell viability.

In addition to these molecular effects of polyQ–Htt aggregates on Nups, polyQ–Htt dose- and age-dependent morphological changes of the NE also occur in HD cell models with perinuclear polyQ–Htt accumulation, in transgenic animal models, and in postmortem HD brain (46, 176). Together these findings show that polyQ–Htt-mediated NCT disruptions are a common phenotype in HD (149, 179).

### NCT Impairments in Normal Aging

Deficits in NCT have not only been linked to age-related neurodegenerative diseases but also appears to be gradually impaired in normal physiological aging (144). The correct assembly, maintenance, and repair of NPCs, which are crucial for cellular health and integrity (145), ask for intact protein homeostasis, a process that is known to be progressively failing during aging. In dividing cells, NPCs disassemble during mitosis and rearrange afterwards in the newly formed cells (180). Rempel et al. showed that NPC quality control is compromised in aging mitotic cells, which results in decreased NPC function and impaired transcription factor shuttling (181). The maintenance of NPCs in long-lived post-mitotic cells, such as neurons, is provided through the renewal of individual NPC subcomplexes, whereby scaffold Nups remain assembled and installed in the NPC during the entire cellular life span (144, 182). These Nups have one of the highest protein lifetimes of organisms; however, they therefore also contribute to NPC vulnerability in advanced age, when molecular damage has accumulated over time. Indeed a study from D'Angelo et al. showed that a subset of scaffolding Nups is oxidatively damaged in aged cells and that the age-related deterioration of NPCs provokes an increase in nuclear permeability accompanied by leakage of cytoplasmic proteins into the nucleus (182).

### NCT Problems Related to Nuclear Import Factors

Nuclear import factors do not only mediate active transport of biomolecules through NPCs but also, in some cases, influence the aggregation of their cargo proteins (183–186). The import factors importin-4, importin-5, importin-7, and importin-β were shown to act as chaperones for exposed basic domains of ribosomal proteins, histones, and other cargos that would otherwise easily aggregate in the polyanionic environment of the cytoplasm (183). A loss of chaperone activity, for example, due to the decrease of import factor RanBP17 with cellular age can lead to NCT impairment, as shown by comparative transcriptomics in fibroblasts and corresponding induced neurons from differently aged donors (187).

Nuclear import factors have also been shown to reverse aberrant liquid–liquid phase separation (LLPS) of proteins and to disaggregate insoluble protein aggregates occurring in neurodegenerative diseases (97, 149, 150). For example, importin-α together with karyopherin-β can disassemble TDP-43 aggregates (150). TNPO1 (= karyopherin-β2) suppresses FUS LLPS and stress granule association (184, 185), whereby disease-linked mutations in the FUS-NLS impair TNPO1 chaperoning and enhance FUS aggregation (185).

## Therapeutic Approaches for Nct Failure

Despite major evidence for NCT problems in ALS, FTD, HD, and AD, up to now no therapeutic approach targeting nuclear transport deficits exists for neurodegenerative diseases. Major challenges in developing therapeutic strategies are given not only by the high molecular and structural complexity of the NPC but also by the importance of NCT for virtually all cellular processes: an intact nucleocytoplasmic trafficking of RNA and proteins is essential to change the transcription profile of a cell, for example, as a response of changes in cellular, substrate, or chemical environment; the NCT of biomolecules is both at the end of all signaling cascades and at the beginning of all cellular responses (40, 41, 188). Finding ways to rescue NCT disruption in neurodegenerative proteinopathies thus holds a tempting new opportunity to prevent neuronal death in these diseases but is also a great challenge.

In the recent years, small molecule nuclear export inhibitors were used with some success for therapeutically targeting nucleocytoplasmic export in cancer and viral disease therapies; however, the lack of compounds that inhibit the nuclear transport of specific cargos compromises the development of therapeutic strategies (153, 189). Disease-induced NPC disruptions often cause an imbalance in the nucleocytoplasmic gradient of NTFs, transcription factors, nuclear proteins, and RNA, which in principle can, to some extent, be reverted by either increasing or inhibiting nuclear import or export (97, 150). For example, it has been shown that inhibition of nuclear import rescues the polyQ–Htt toxicity in a yeast model (190), whereas inhibition of nuclear export was neuroprotective in a cell model of ALS (43, 45, 147, 191). Structure-based design of inhibitors that target exportin-1/CRM, the major receptor for the export of proteins out of the nucleus, yielded selective inhibitors of nuclear export (KPT-350, KPT-335, and KPT-276) that also proved successful in preclinical models. However, due to the broad range of molecular cargos shuttled out of the nucleus with the help of exportin-1/CRM1, off-target effects and potential toxicity remain as important issues when targeting this pathway (153, 192). A phase 1 safety trial using the exportin1 inhibitor XPO1 has recently been launched to investigate the safety and beneficial vs. the off-target effects of exportin-1 inhibition in ALS patients (193). This trial will hopefully also clarify whether targeting nucleocytoplasmic export will be sufficient to alleviate pathological neuronal death in the human brain.

In any case, since differences exist in the molecular and the cellular disease mechanisms between neurodegenerative diseases and aging seems to play a role for NPC function as well, physiological and disease-specific NPC/NCT alterations need to be investigated in more detail. For the development of tau-targeted NCT-based therapies, we are still at the very beginning, and systematic analyses of tau–NPC interactions and their downstream effects are needed.

## Conclusion

**Figure 2:**
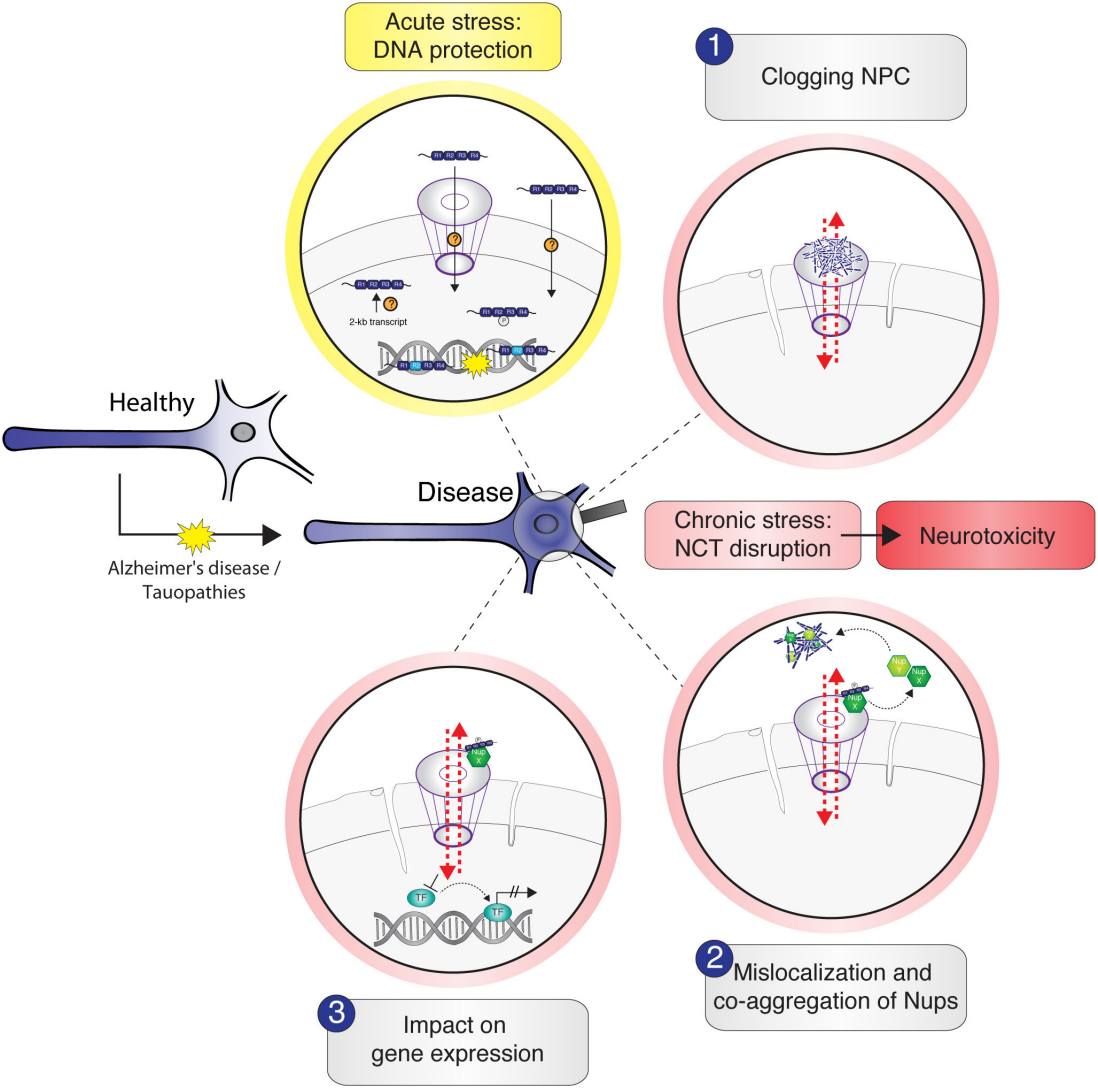
Schematic illustration of potential tau–nucleus interactions leading to nucleocytoplasmic transport (NCT) impairment in tau-related neurodegeneration. Under physiological conditions, cytosolic soluble tau is mainly localized to the neuronal axon to stabilize the microtubules. In stress conditions and in the context of neurodegenerative diseases like Alzheimer's disease and tauopathies, tau mislocalizes from the axon into the somatodendritic compartment where it gets in close proximity to the nucleus. Acute stress, for example, via heat shock, transiently increases the amount of intranuclear tau, either by active transport of tau through the nuclear pore complexes (NPC) or other unknown import mechanisms or by the enhanced expression and/or local translation of nuclear tau transcripts. Nuclear tau binds and stabilizes DNA during the time of insult, undertaking a DNA-protective role. Under persistent stress—as in the context of neurodegenerative diseases—the amount of hyperphosphorylated tau in the soma increases further and leads to different possible scenarios of tau-induced NCT disruption, which are accompanied with nuclear envelope abnormalities (e.g., invaginations) and result in neurotoxicity: (1) soluble and/or aggregated tau binds and thereby clogs the nuclear pore, resulting in cargo transport inhibition; (2) tau interacts with specific Nups of the NPC, leading to NPC disassembly and sequestration of Nups from the NPC into the cytosol, resulting in nuclear pore leakiness and co-aggregation of cytoplasmic Nups with tau; (3) somatodendritic tau interacts with Nups that under physiological conditions would associate with transcription factors to regulate gene expression. These interactions “distract” Nups and thereby indirectly affect gene expression.

Whereas, the presence of tau inside the nucleus has been reported for several years, tau interactions with the NE and their consequences for neuronal NCT were described only recently. NCT impairment and concomitant neurotoxicity in tau-associated NDDs could result from different (hypothetical) scenarios of tau interactions with nuclear pore complexes, with individual Nups, or with NTRs (Figure 2). However, to decipher the physiological role of tau in chromatin regulation and the consequences of tau–NPC interactions in pathophysiological conditions, we need to systematically identify tau–NPC, tau–NTR, and tau–chromatin interactions in order to understand the molecular mechanisms and the (dys) functional role of tau's interactions with the nucleus.

Furthermore, we want to acknowledge that the occurrence of NCT problems in protein aggregation diseases (including tau-related ones) is a rather young observation; however, it yet is a new interesting emerging field in disease neurobiology that offers complementary interpretations to established disease mechanisms associated with neuronal protein aggregation.

## Author Contributions

LD wrote the first draft of the manuscript. SW and LD edited the manuscript to the final version. All authors contributed to the article and approved the submitted version.

## Conflict of Interest

The authors declare that the research was conducted in the absence of any commercial or financial relationships that could be construed as a potential conflict of interest.
